# Recovery and Stabilization of Anthocyanins and Phenolic Antioxidants of Roselle (*Hibiscus sabdariffa* L.) with Hydrophilic Deep Eutectic Solvents

**DOI:** 10.3390/molecules25163715

**Published:** 2020-08-14

**Authors:** Oscar Zannou, Ilkay Koca, Turki M. S. Aldawoud, Charis M. Galanakis

**Affiliations:** 1 Department of Food Engineering, Ondokuz Mayis University, Samsun 55139, Turkey; 2 College of Science, King Saud University, Riyadh 11451, Saudi Arabia; tdawoud@ksu.edu.sa (T.M.S.A.); cgalanakis@chemlab.gr (C.M.G.); 3 Research & Innovation Department, Galanakis Laboratories, 73131 Chania, Greece; 4 Food Waste Recovery Group, ISEKI Food Association, 1190 Vienna, Austria

**Keywords:** roselle, deep eutectic solvent, anthocyanin, antioxidant, response surface methodology, green chemistry

## Abstract

Deep eutectic solvents (DESs) have got huge interest as new green and sustainable solvents for the extraction of bioactive compounds from plants in recent decades. In the present study, we aimed to investigate the effectiveness of hydrophilic DES for the extraction of anthocyanin and polyphenol antioxidants from Roselle. A natural hydrophilic DES constituted of sodium acetate (hydrogen bond acceptor) and formic acid (hydrogen bond donor) designed to evaluate the total phenolic compound (TPC), total flavonoid (TFC), total anthocyanin (TACN), 2,2-diphenyl-1-picrylhydrazyl (DPPH) radical scavenging and ferric reducing antioxidant power (FRAP) values of Roselle. Distilled water, 70% ethanol, and 80% methanol used as conventional solvents for comparison. The results indicated that the DES prepared in molarity ratio (SAFA_m_) was the most efficient. Subsequently, this prominent DES selected for the optimization and the optimum extraction conditions were 1:3.6 molarity ratio, 0% additional water, and 10 mL solvent. TPC, TFC, TACN, FRAP, and DPPH radical scavenging at the optimum point were 233.26 mg GAE/g, 10.14 mg ECE/g, 10.62 mg D3S/g, 493.45 mmol ISE/g, and 343.41 mmol TE/g, respectively. The stability tests showed that anthocyanins were more stable in SAFA_m_. These findings revealed that SAFA_m_ is an effective green solvent for the extraction of polyphenols from various plants.

## 1. Introduction

Roselle (*Hibiscus sabdariffa* L.) is one of the most popular species belonging to the Malvaceae family. They are highly desired by consumers, for containing several nutrients and phytochemical compounds that improve health. Roselle is essential for the prevention and treatment of hypertension, hepatitis, cardiovascular diseases, atherosclerosis, and diabetes [1,2,3,4]. The extracts had powerful effects on reducing high cholesterol, preventing cancer and hepatitis, and inhibiting pathogen microorganisms’ growth [2,5]. Roselle’s red calyces are processed to juice and tea, or incorporated in confectionary (e.g., chocolate) and dairy products as natural flavoring, and coloring agents. The calyces remain as the primary by-product and provide natural antioxidants and odorants [6,7,8].

Food and plant bioactives are nowadays very important in supporting the immune system within the COVID-19 pandemic era [9]. Roselle calyces contain bioactive compounds that are useful for human health such as gallic, syringic, caffeic, chlorogenic, neochlorogenic, cryptochlorogenic, ferulic acids, quercetin, naringenin, rutin, delphinidin 3-o-sambubioside, cyanidin 3-o-sambubioside, delphinidin-3-glucoside, cyanidin-3-kaempferol, and their glycosides with 5-(hydroxymethyl) [10,11,12,13,14,15]. Among the polyphenols present in Roselle calyces, anthocyanins are the most abundant flavonoid. The anthocyanins present in the Roselle have many pharmacological properties. They have demonstrated nephroprotective and antioxidative effects [16]. They could also be useful against ultraviolet radiation-induced cutaneous and ocular diseases [17]. Therefore, it is beneficial to extract polyphenols, especially anthocyanins, from Roselle.

The recovery of compounds from natural sources is typically conducted using the 5-Stages Universal Recovery Process: Pre-treatment, separation of macro- and micro-molecules, extraction, purification and product formation [18]. Among these stages, the third one (extraction) is the most essential one [19,20]. Most of the conventional extraction methods have drawbacks to require long extraction times and consumption of a high amount of organic solvent as well as apparent thermal degradation of phytochemicals [21,22,23]. The most common organic solvents used for the extraction of polyphenols are associated with possible inflammation, volatility, explosivity, toxicity, and environmental pollution [24]. Therefore, the development of environmentally friendly solvents with low toxicity and cost, as alternatives to the conventional organic solvents, became mandatory [25]. The extraction of phytochemicals with hydrotropic solvents is becoming the new trend [26,27].

Deep eutectic solvents (DESs) generated from the blend of two or more components consisting of hydrogen bond donor (HBD) and hydrogen bond acceptor (HBA), and establishing a supramolecular structure with hydrogen bonds [28]. DESs have much lower melting points than that of either single components [29,30]. They are mixtures of easily to be prepared, natural, renewable, non-toxic, and cheaper components [25,31,32]. They are also characterized by thermal and chemical stability, high solubility, low volatility, biodegradability, low vapor pressure, flexibility, strong biocompatibility, and designability [33,34]. Furthermore, DESs are worthwhile for the extraction of polyphenols since they provide higher extractability performance than the conventional solvents. They have been successfully employed for the extraction of polyphenols in many products [35]. Despite the suitability of DESs for polyphenols extraction, their high viscosity became a serious roadblock affecting their application and yield.

The high viscosity of DESs constitutes severe obstacles for their implementation in industry, even though the fluidity of these solvents might be increased by adding some quantities of water [28]. Therefore, the hydrophilic DESs have been developed, and the effects of water on their structure, as well as properties have been investigated [35,36,37,38,39,40]. For the extraction of polyphenolic compounds, diverse water contents either as a molar ratio or as a percentage have been reported [30,41,42,43,44,45,46]. Some authors researched the extraction of anthocyanins and polyphenolic antioxidants from Roselle calyces using conventional and citric acid-based DESs [14,17,47,48,49]. Furthermore, to the best of our knowledge, limited research has been published about the use of ultrasound-assisted extraction (UAE) for the recovery of polyphenolic antioxidants from Roselle calyces. In the present study, deep eutectic solvent-based ultrasound-assisted extraction of polyphenolics with antioxidant capacity from Roselle was investigated for the first time. The main aim of this study was to recover the maximum anthocyanin from Roselle using DES. For this purpose, a natural hydrophilic deep eutectic solvent (DES) designed and prepared using sodium acetate as HBA and formic acid as HBD. The effect of water addition on the extraction of polyphenols of Roselle was determined. The extraction using distilled water, aqueous ethanol and methanol as conventional solvents carried out for comparison. Also, the most prominent DES was chosen for the optimization using Box Behnken design along with Response Surface Methodology and for the stability of anthocyanin in DES.

## 2. Results and Discussion

### 2.1. Characterization of DESs

#### 2.1.1. Viscosity

The DESs prepared in molar and molarity ratios were encoded as SAFA_0_ and SAFA_m_, respectively. Each DES was characterized by high viscosity ranged between 6.15 and 3651.6 mPa.s (Table 1). The presence of carboxyl groups and alkyl chains causes extremely high viscosity [35,50]. The pure SAFA_0_ (0% water content) was semi-solid at room temperature and displayed an extremely high viscosity of 3651.6 mPa.s. This high viscosity is associated with the H bonding network generated by the combination of sodium acetate and formic acid in eutectic terms. The addition of water decreased DES viscosity. The application of water in DESs has been reported to weaken and disintegrate the DESs nanostructure [35,40]. In the present study, the application of water decreased the viscosity to 61.6 mPa.s at 20% (*v*/*v*), 17.36 mPa.s at 40% (*v*/*v*), 10.81 mPa.s at 60% (*v*/*v*) and 7.66 mPa.s at 80% (*v*/*v*). The lowest viscosity was recorded in the DES prepared in molarity ratio (SAFA_m_), suggesting that it contained more water. Therefore, the interaction between sodium acetate and formic acid might be the lowest since high water concentration in DESs reduces the intermolecular and intramolecular reactions between the components constituting DESs [39,40,51].

#### 2.1.2. FTIR

**Figure 1:**
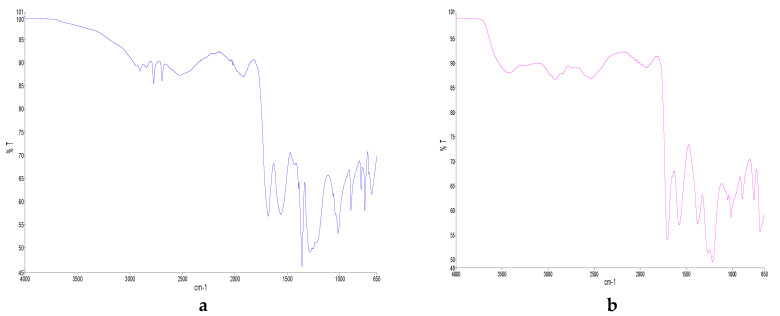

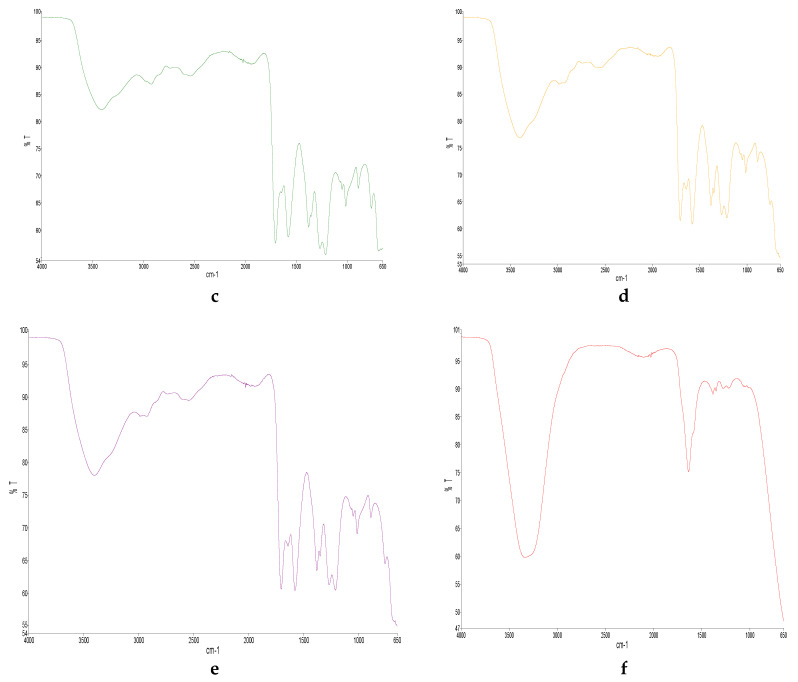
FTIR diagram of (**a**) SAFA_0_, (**b**) SAFA_20_, (**c**) SAFA_40_, (**d**) SAFA_60_, (**e**) SAFA_80_ and (**f**) SAFA_m_.

The FTIR spectra of all the DESs studied were shown in Figure 1. The peaks at 2800 and 2650 cm^−1^ (Figure 1a) were assigned to C-H stretching frequencies associated with formic acid and sodium acetate, respectively. The frequencies at 1650 and 1550 cm^−1^ were related to C=O of formic acid and sodium acetate, respectively. The stretching of C-O at 1350 cm^−1^, CH bending at 1250 and 1000 cm^−1^ and stretching of C-C at 900 cm^−1^ were used to recognize sodium acetate. From spectroscopy data, it can be assumed that the strong COOH...COOH bonds in sodium acetate and formic acid were broken down during the formation of sodium acetate and formic acid mixture to produce new strong intramolecular COOH bonds. This explains the low melting point and high viscosity of the pure SAFA_0_ [35].

After the addition of water, the stronger bonding between HBD and HBA was affected and weakened with the appearance of O-H (Figure 1). The absence of O-H in SAFA_0_ suggested that the mixture was too anhydrous. The stretching of O-H at 3400 cm^−1^ appeared progressively in DESs, got higher as water content increased and became stronger in SAFA_m_ (Figure 1b–e). Similar phenomena have been reported in choline chloride and oxalic acid, as well as in choline chloride and glycol-based DESs, and might be due to alternative possibilities for H bonding in DES upon the introduction of water [35,40]. Hammond et al. (2017) have revealed that the introduction of a small quantity of water affected immensely the nanostructure of DES. However, FTIR analysis showed that the shape of spectra was kept after addition of 80% water. This indicates the tolerance of DES structure [35,52]. Nonetheless, C-H stretching weakened upon addition of water and disappeared completely in SAFA_m_.

### 2.2. Evaluation of DESs Efficiency

In the present study, sodium acetate:formic acid (1:2 molar ratio) was employed as a natural DES and coded as SAFA_0_ to extract polyphenolic compounds, especially anthocyanins and to evaluate the antioxidant activity of Roselle calyces. The major roadblock of DESs which limits their applications and efficiency for phytochemicals is the high viscosity. This fact reduces consequently the mass transfer and solubility of phenolic compounds [42,53,54]. To alleviate this constraint, DES sodium acetate:formic acid was diluted with 20, 40, 60 and 80% of water and encoded SAFA_20_, SAFA_40_, SAFA_60_, and SAFA_80_, respectively. Furthermore, another sodium acetate:formic acid-based DES was designed in molarity ratio 1:2 (SAFA_m_), following the studies by the authors in [55], who have suggested the DESs betaine:tartaric acid and betaine:citric acid prepared in molarity ratio. In addition, for the purposes of comparison, distilled water, 70% ethanol and 80% methanol were used as conventional solvents. Roselle calyces were extracted with these solvents using ultrasound. The results of total phenolic (TPC), total flavonoid (TFC), total anthocyanin (TACN), DPPH radical scavenging and FRAP were shown in Table 2.

Globally, when compared the efficiency of the solvents used in this study, the DESs were more efficient than the conventional solvents for the extraction of Roselle polyphenolics, except SAFA_0_ and SAFA_20_ (Table 2). The lowest results provided by SAFA_0_ and SAFA_20_ were due to their extremely viscosity (Table 1). The viscosity of DESs hinders the mass transfer in the extraction matrix, decreasing the extractability performance of DESs [34,41,44,56]. The highest TPC was detected with SAFA_m_, following SAFA_80_, SAFA_60_, SAFA_40_ and SAFA_20_, respectively. The TPC obtained with SAFA_m_ was 43.11, 43.68 and 43.68% higher than the values obtained with distilled water, 70% ethanol and 80% methanol, respectively. Similar results have been figured out in rosemary, where TPC obtained with acid-based DESs was 15–18% higher than ethanol [42]. Likewise, the highest TFC, TACN, DPPH radical scavenging and FRAP were determined with SAFA_m_ and SAFA_80_. SAFA_m_ yielded 1.22, 3.53 and 4.99-fold higher TFC than 70% ethanol, 80% methanol and distilled water, respectively. Similarly, the TFC has been well-extracted from saffron with lactic acid-based DES [57]. However, sodium acetate:formic acid provided higher yields of TPC of Roselle when compared to the yields of citric acid:glycerol (24.79 mg GAE/g) and citric acid:ethylene glycol (36.31 mg GAE/g) [49], except SAFA_0_. In addition, TACN obtained with SAFA_m_ was 1.03, 1.09 and 1.23-fold higher than 70% ethanol, distilled water, and 80% methanol, respectively. Meanwhile, the values of TACN determined in this study were in accordance with the findings previously detected in Roselle with citric acid:glycerol (5.44 mg C3G/g) and citric acid:ethylene glycol (9.36 mg C3G/g) [49]. The high extraction yield of SAFA_m_ is associated with the multiple hydrogen-bonding and low viscosity. The carboxyl group (-COOH) of formic acid presented more interactions between hydrogen-bonding [37]. The high efficiency of acid-based DESs for the extraction of polyphenolics have been proved and told to due to the H-bonding interactions among DES components and the low viscosity [57,58,59,60,61]. SAFA_m_ was found to be an ideal medium for the extraction of Roselle polyphenolics, allowing a high mass transfer and solubility of anthocyanins.

### 2.3. Optimization of DES and Extraction Conditions

#### 2.3.1. Model Analysis

The responses (total anthocyanin, total phenolic compound, DPPH radical scavenging, FRAP and total flavonoid values) and the coded independent factors of experimental points were shown in Table 3. A total of 17 experimental points was investigated. TPC was ranged from 105.81 to 233.20 mg GAE/g. The TFC values were found between 3.78 and 10.13 mg ECE/g, while TACN values were ranged from 6.50 and 10.90 mg D3S/g. The values of FRAP were found between 305.86 and 493.88 mmol ISE/g, while DPPH radical scavenging values were ranged from 222.05 and 372 mmol TE/g. The highest TPC, TFC, TACN, FRAP and DPPH radical scavenging were detected at run 7 (SAFA_m_ 1:4, 0% additional water and 25 mL solvent). While, the lowest TPC and FRAP values were observed at run 5 (SAFA_m_ 1:2.5, 30% additional water and 25 mL solvent), and the lowest DPPH radical scavenging at run 11 (SAFA_m_ 1:2.5, 30% additional water and 25 mL solvent). Likewise, the lowest TFC and TACN values were detected in the run 4 (SAFA_m_ 1:2.5, 60% additional water and 40 mL solvent) and run 14 (SAFA_m_ 1:2.5, 30% additional water and 25 mL solvent). The lowest values of the responses were figured out at the runs where DESs were prepared with additional water ranged between 30 and 60%. At these experimental points, the viscosity was very low, indicating that the DES might lose its intrinsic characteristics and become a simple aqueous solution. It has been reported that a very low viscosity induce less hydrogen-bonding and decrease the extraction yield of phytochemical compounds [37,62]. The highest values were detected in the runs where DES did not require additional water. These findings revealed the importance of introduction of water in DES and indicated that the optimum DES will not have too low or too high viscosity. Moreover, the highest responses were obtained with molarity ratio 1:4.

For the analysis of the optimization models, the regression coefficients were indicated at the least square for the second-order quadratic polynomial models. The stepwise option of response surface methodology was used to eliminate the non-significant parametric values [63]. The reduced second-order models in terms of actual factors for the responses such as TPC, TFC, TACN, FRAP and DPPH radical scavenging values of Roselle calyces as a function of molarity ratio (X_1_), additional water (X_2_) and solvent to solid ratio (X_3_) were obtained as follows:

TPC = 110.22 + 11.63X_1_ − 20.25X_2_ − 15.13X_3_ + 13.00X_2_X_3_ + 48.28X_1_^2^ + 49.03X_2_^2^ + 50.28X_3_^2^ (1)

TFC = 7.60 − 1.75X_3_ − 0.75X_1_X_3_ − 0.75X_2_X_3_ + 0.75X_2_^2^ − 2.05X_3_^2^ (2)

TACN = 6.86 + 0.23X_1_ − 0.60X_2_ + 0.08X_3_ − 0.91X_1_X_2_ + 0.72X_1_^2^ + 1.07X_2_^2^ + 0.62X_3_^2^ (3)

FRAP = 314.60 − 30.63X_2_ − 26.13X_3_ + 5.50X_1_X_2_ + 43.33X_1_^2^ + 62.58X_2_^2^ + 28.58X_3_^2^ (4)

DPPH = 227.60 + 16.75X_1_ − 13.50X_2_ − 8.50X_3_ − 12.50X_1_X_2_ − 24.50X_1_X_3_ + 10.50X_2_X_3_ + 21.70X_1_^2^ + 49.50X_2_^2^ + 29.20X_3_^2^ (5)

These equations showed up the response patterns for individual measurement and intricacy of sceneries. The higher and positive parametric values translate the more significant the weight of the governing variable is [64]. The results of the ANOVA showing the effects of different parametric values on the responses along with R2, adjusted R2, predicted R2, adequate precision and coefficient of variance (CV) were assigned in Table 4. The model developed for TPC, which provided higher R^2^ (0.9880) and adjusted R^2^ (0.9725) was significant at *p* ˂ 0.0001. The R^2^ and adjusted R^2^ values displayed a good closeness, indicating that there was strong conformity between the experimental findings and predicted values. The CV of 4.71% revealed that the model was better reproducible for TPC, as CV expresses the standard deviation in percentage. The adequate precision ratio of 22.75 indicates that the model can be employed to explore the design because an adequate precision ratio higher than 4 is desirable. The lack of fit which was non-significant indicated that the developed model was a good fit at F value 1.70 (*p* ˂ 0.3046). The predicted R^2^ of 0.8841 for TPC is in reasonable agreement with the adjusted R^2^ of 0.9725; i.e., the difference is less than 0.2. The model developed for TFC was significant at F value 30.84 (*p* ˂ 0.0001) and had satisfactory R^2^ (0.9655), adjusted R^2^ (0.9211), CV (6.45%) and adequate precision (20.41). This model presented a non-significant lack of fit, conferring a good fitness to the model. Furthermore, the R^2^ and adjusted R^2^ of the model developed for the TFC were very close and higher which implicated there was great conformity between the experimental and predicted values. The predicted R^2^ of 0.9003 for TFC is in reasonable agreement with the adjusted R^2^ of 0.9438; i.e., the difference is less than 0.2. For TACN, the model was significant at F value 21.77 (*p* ˂ 0.0003) and had desirable R^2^ (0.9655), adjusted R^2^ (0.9211), CV (3.63%) and adequate precision (15.84). nonetheless, the predicted R^2^ of 0.6098 is not as close to the adjusted R^2^ of 0.9211 as one might normally expect. This may indicate a large block effect or a possible problem with the model and/or data. This model presented non-significant terms for lack of fit, indicating that the model was a good fit and adequate, since there was great conformity between the experimental and predicted values.

In relation to the model developed for DPPH radical scavenging, acceptable R^2^ (0.9864), adjusted R^2^ (0.9688), CV (2.57%) and adequate precision (21.06) with significant terms at F value 58.26 (*p* ˂ 0.0001). The predicted R^2^ of 0.8163 for DPPH radical scavenging is in reasonable agreement with the adjusted R^2^ of 0.9688 and a non-significant lack of fit approved the adequacy of the model. With respect to the model generated for FRAP, significant terms were found at F value 32.78 (*p* ˂ 0.0001) with higher R^2^ (0.9768), adjusted R^2^ (0.9470), CV (3.10%) and adequate precision (18.13). These results indicated that there was strong conformity between the experimental findings and predicted values, and the model adequate and reproducible for FRAP values of Roselle calyces. In addition, the model generated for FRAP was good fit by providing a non-significant lack. These analytical findings proved that the parametric values for TPC, TFC, TACN, DPPH radical scavenging and FRAP values by response surface methodology can be used for the prediction and optimization stages [64]

#### 2.3.2. Effects of Independent Variables on the Responses

The linear, quadratic and interactions terms of the models on TPC, TFC, TACN, FRAP and DPPH radical scavenging of Roselle calyces were presented in Table 4. The linear and quadratic terms had significant effects on TPC. However, the interaction terms of additional water and liquid to solid ratio had no significant effects on TPC with F values of 9.43 (*p* ˂ 0.0180). The parametric value having the most impactful effects on TFC was linear term of liquid to solid ratio with F value of 97.35 (*p* ˂ 0.0001), followed by the interaction terms of molarity ratio/liquid to ratio and additional water/liquid to solid ratio with F value of 10.86 (*p* ˂ 0.0132), and the quadratic terms of additional water with F value 9.96 (*p* ˂ 0.0160), and liquid to solid ratio with F value 85.42 (*p* ˂ 0.0001), respectively. All linear terms and quadratic effects on TACN. Moreover, the interaction term of molarity ratio and additional water provided significant on TACN at F value 38.80 (*p* ˂ 0.0004). As shown in Table 4, all the linear, interaction and quadratic units showed significant effects on DPPH radical scavenging values of Roselle calyces Likewise, the linear units having the most impactful effects on FRAP were found to be additional water at F value 82.80 (*p* ˂ 0.0001), followed by liquid to solid ratio at F value 16.29 (*p* ˂ 0.0050), while the most impactful quadratic units were additional water F value 100.05 (*p* ˂ 0.0001), followed by molarity ratio F value 47.96 (*p* ˂ 0.0002) and liquid to solid ratio F value 20.86 (*p* ˂ 0.0026). In addition, the interaction of molarity ratio and additional water displayed significant effect on FRAP F value 15.17 (*p* ˂ 0.0059).

**Figure 2:**
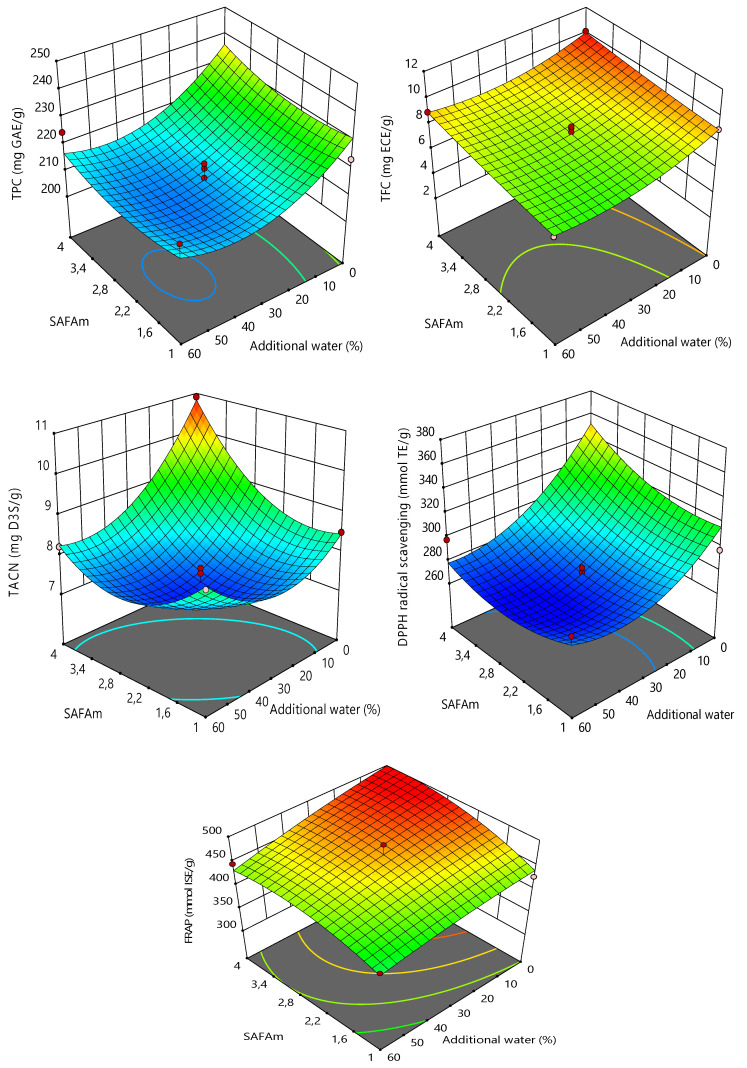
3D response surface plots showing the effect of independent variables on the responses.

The three-dimensional (3D) response surface plots of the models were used to interpret the effects of interactions between the variables on the responses (Figure 2). The 3D response surface plots showed that the TPC, TFC, TACN, FRAP and DPPH radical scavenging values were strongly influenced by molarity ratio and additional water. From the figures, it can be observed that an increase in molarity ratio induced a rapid increase in TPC, TFC, TACN, FRAP and DPPH radical scavenging values. This can be explained because the carboxyl group (-COOH) and sodium cation (-Na^+^) from sodium acetate would combine with the carboxyl group (-COOH) or the hydroxyl group (-OH) of formic acid. With the lower molarity ratio of formic acid, there are many specific groups from HBA, which could not bond with the specific groups of HBD, leading to the precipitation of DESs [37,53]. Nonetheless, the effects of molarity ratio were moderate when compared to the effects of additional water.

As demonstrated in Figure 2, the addition of water led to a decrease in TPC, TFC, TACN, FRAP and DPPH radical scavenging values. The highest results were recorded with 0% additional water. However, a slight increase in TACN was observed in 40–60% of additional water. 0% additional water at the maximum response values might be explained because the DESs used in the present study were prepared as molarity terms. The hydrophilic DESs are greatly recommended for the isolation of bioactive compounds, facilitating their diffusion and solubility [61,62]. SAFA_m_ was yet hydrophilic, implying that the further addition of water could be harmful to the DES and decrease the extraction performance. The hydrophilic DESs are easy to use, reduce the viscosity and preparation cost and increase polyphenolic extraction yields, nonetheless, a certain of amount of water could not be overpassed [30,33,57,65]. Adding more water weakens the interactions between the hydrogen-bonding of different components, affecting the extraction yield [37].

#### 2.3.3. Multi-Response of the Optimization Process

The response surface methodology was performed to optimize TPC, TFC, TACN, FRAP and DPPH radical scavenging values. The optimum conditions were 1:3.6 molarity ratio, 0% additional water and 10 mL solvent ratio. Under these optimum conditions, the predicted TPC, TFC, TACN, FRAP and DPPH radical scavenging values were 239.30 mg GAE/g, 10.45 mg ECE/g, 10.93 mg D3S/g, 503.83 mmol ISE/g and 355.42 mmol TE/g, respectively. For confirmation, further analyses were performed under optimum conditions. The results were presented as 233.26 mg GAE/g, 10.14 mg ECE/g, 10.62 mg D3S/g, 493.45 mmol ISE/g and 343.41 mmol TE/g for TPC, TFC, TACN, FRAP and DPPH radical scavenging, respectively.

### 2.4. Efficiency of Response Surface Methodology on Total Anthocyanin

**Figure 3:**
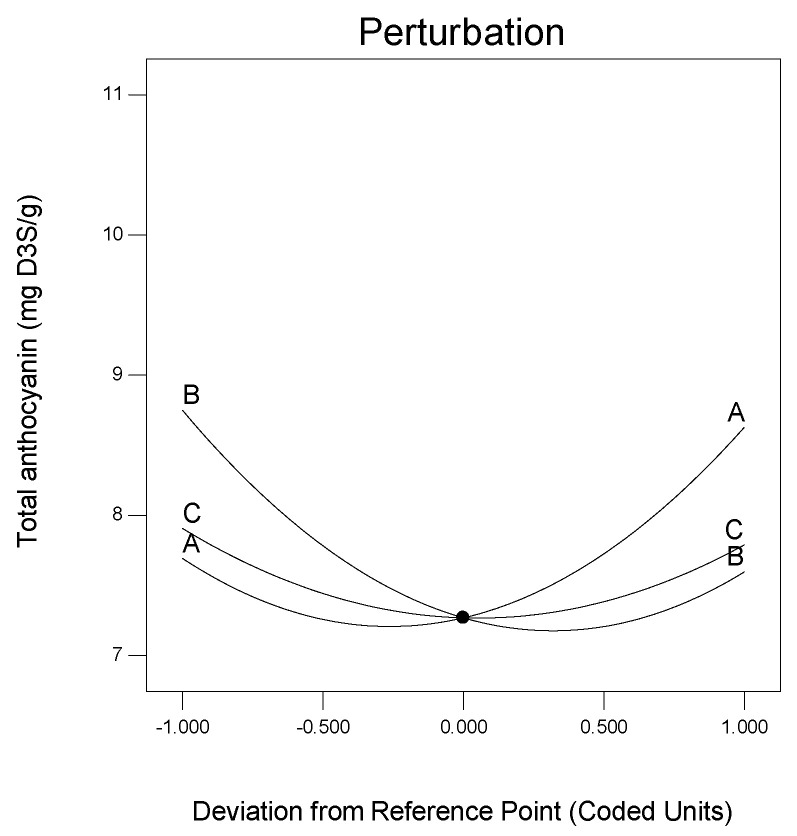
Perturbation graphic showing the effects of variables on total anthocyanin.

The TACN of Roselle calyces in SAFA_m_ increased from 6.90 to 10.93 mg D3S/g after the application of response surface methodology. These values were found to be higher than the total anthocyanin previously detected in Roselle calyces which has been ranged between 1.22 and 2.3 mg/g [16,48]. This is due not only to the origin and variety of the plant material but mostly to the use of DESs and UAE for the extraction. The DESs are natural and eco-friendly solvents which are prominent for the extraction of bioactive compounds including anthocyanins [41,44,53,56,58,60,66]. The DES (sodium acetate:formic acid) used in the present study extracted better anthocyanin from Roselle calyces. Furthermore, the application of UAE combined with DESs is well-adapted and accelerates the mass transfer of the analytes [67,68,69]. The effects of molarity ratio, additional water and solvent ratio on the response anthocyanin was well-represented by perturbation graphic generated using response surface methodology (Figure 3). As can be observed, the total anthocyanin of Roselle calyces behaved differently with variables. The curvature observed indicated that TACN was sensitive to all the variables. However, the TACN was mostly affected by molarity ratio and additional water, as these variables showed great variations (Figure 3).

### 2.5. Stability of Antioxidant Properties of Roselle in SAFA_m_

The polyphenols recovered from different natural sources find applications in foods [70] and cosmetics [71] where stability is a critical factor for their successful implementation. The optimum experimental conditions figured out with response surface methodology were used to evaluate the heat and storage stabilities of TPC, TFC, TACN, DPPH radical scavenging and FRAP of Roselle.

#### 2.5.1. Thermal Stability

**Figure 4:**
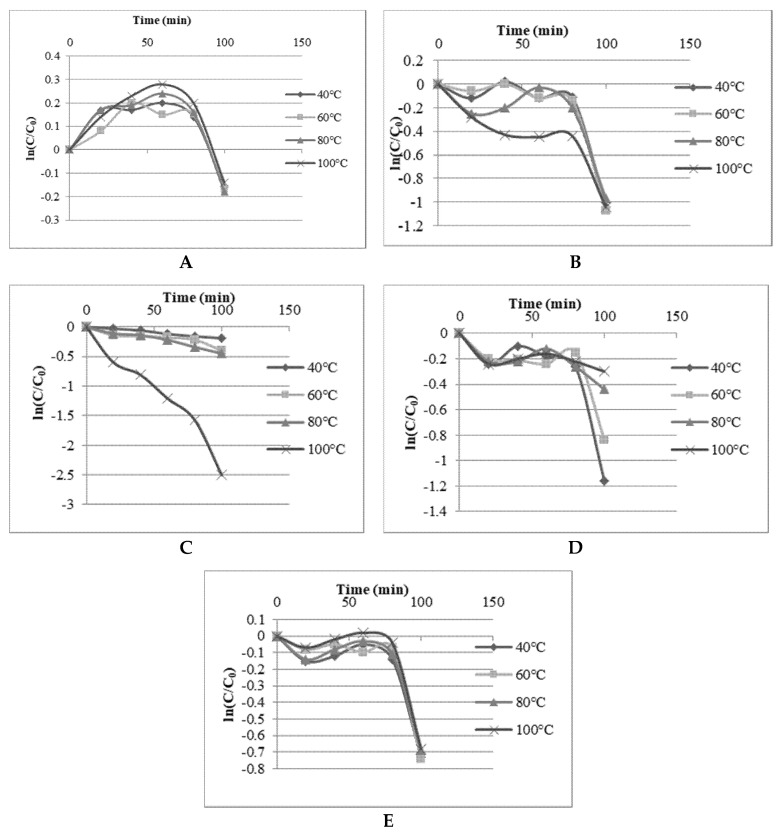
Stability of TPC (**A**), TFC (**B**), TACN (**C**), DPPH radical scavenging (**D**) and FRAP (**E**) values in SAFA.

The heat effects on TPC, TFC, TACN, DPPH radical scavenging and FRAP values of the DES extract of Roselle were determined by applying 40, 60, 80 and 100 °C for 20, 40, 60, 80 and 100 min. The first-order kinetics was determined for the thermal degradation of TPC, TFC, TACN and antioxidant properties of Roselle in SAFA_m_ (Figure 4). The TPC, TFC, DPPH radical scavenging and FRAP values of Roselle extracts showed strong stability in SAFA in all experimental temperatures and time. The thermal degradation started between 60 and 80 min of heating, except at 100 °C which induced a thermal degradation of TPC and TFC earlier at 60 min, and 20 min, respectively. The degradation rate was steeper for all the responses at 100 °C and 80 min. These findings were in agreement with the previous thermal degradations of TPC, TFC and antioxidant activity reported in elderberry and lavender to be accentuated after 5–90 min at 75–150 °C [72,73,74]. TACN was more stable at 40, 60 and 80 °C in SAFA_m_ but decreased drastically at 100 °C. Similarly, the thermal degradation of anthocyanin in aqueous extracts of Roselle has been reported to be crucial between 60 and 90 °C [75,76]. Generally, the phytochemical compounds with antioxidant properties in Roselle were found more stable in SAFA_m_. The strong thermal stability behavior of polyphenols in SAFA_m_ could be associated with a strong hydrogen network between extracts and DESs components [58]. However, the thermal degradation rate increased by augmenting both temperature and heat time. The first-order reaction constant k and activation energy for isothermal kinetics of anthocyanin degradation were assigned in Table 5. The values k figured out in this study confirmed the influence of temperature and time on anthocyanin. It was observed that k values increased with augmenting temperature from 2.39 × 10^−5^ at 40 °C to 49.96×10^−5^ s^−1^ to 100 °C for times varying between 20 to 100 min. Other authors have also mentioned that k values increased with augmenting temperature and time [14,58,76,77,78]. The activation energy (Ea) was ranged from 36.78 to 49.13 kJ/mol and was slightly less than Ea values reported aqueous extracts of Roselle [75,76] and in blackcurrant juice [78].

#### 2.5.2. Stability of Total Anthocyanin during Storage

**Figure 5:**
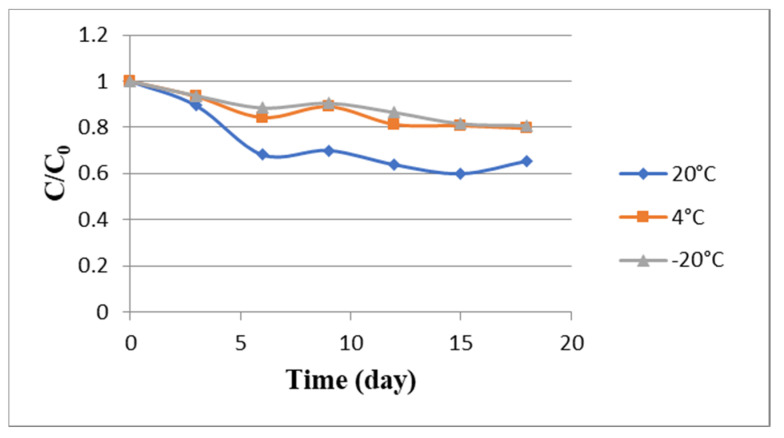
Degradation kinetics of Roselle anthocyanin in SAFA_m_ during storage at different temperatures.

The degradation of anthocyanin in SAFA_m_ was investigated during eighteen days at room temperature (20 °C), 4° and −20 °C, and the results were represented in Figure 5. The concentrations of anthocyanin decreased slowly in SAFA_m_ with time at all temperatures. However, this decrease was steeper at higher temperature (20 °C). The degradation rates were 40.10, 20.46 and 19.25% at 20 °C, 4 °C and −20 °C, respectively. The linear relation between the logarithm of total anthocyanins and time indicated a first-order kinetic for Roselle anthocyanin degradation in SAFA_m_. This is in agreement with the previous investigations in aqueous extracts of Roselle [14,75]. The anthocyanin degradation was too slow in SAFA_m_, especially after 12 days, showing the strength of intramolecular and intermolecular reactions occurred between solute and SAFA_m_. Nonetheless, the storage temperature had an influence on anthocyanin degradation (Table 6). As can be seen, the values of k varied from 1.29 × 10^−7^ to 2.52 × 10^−7^ s^−1^ at −20 °C, from 1.47 × 10^−7^ to 3.47 × 10^−7^ s^−1^ at 4 °C and from 2.73 × 10^−7^ to 7.41 × 10^−7^ s^−1^ at 20 °C. During the storage, the values of the first-order rate constant (k) of the model increased 5.74-fold. Furthermore, the values of t_1/2_ were ranged 0.94 × 10^6^ to 5.39 × 10^6^ s and the highest t_1/2_ values were found at −20 °C and 4 °C, respectively. k values detected in this study were low and t_1/2_ values were higher when compared to k and t_1/2_ values reported in previous studies [14,76]. This indicated that SAFA_m_ was more anthocyanin-protective during the storage when compared to water.

## 3. Materials and Methods

### 3.1. Plant Material

Roselle collected from the experimental farms of the University of Agriculture of Kétou, located in Kétou province, Benin Republic. The fresh Roselle calyces were sun-dried for 7 days. The dried calyces were packed into cleaned and sterilized brown bottles and kept at 4 °C for further use. Prior analysis, the dried calyces were pulverized by a disintegrator (Sinbo, coffee and spice grinder, SCM 2934), sieved, and brown bottle at 4 °C.

### 3.2. Chemicals and Reagents

Distilled water purified by a Millipore-Q system (Millipore Billerica, Massachusetts, USA). Methanol (≥99,8%), 2,2-diphenyl-1-picrylhydrazyl (DPPH), 2,4,6-tris(2-pyridyl)-s-triazine (TPTZ, ≥99.0%), Trolox (97%), sodium nitrite (99–100.5%), hydrochloric acid (36.5–38%), (-)- epicatechin, gallic acid (≥99.0) and sodium carbonate (99.5–100.5%) were bought from Sigma-Aldrich. Ethanol (≥99.9%) purchased from Isolab. Folin-Ciocalteu reagent, aluminum chloride, iron (III) chloride, iron sulfate heptahydrate (≥99.5%) and formic acid (98–100%) brought from Merck. Sodium acetate anhydrous (≥99.0%), glacial acetic acid (99.5%), potassium chloride (≥99.0%), and Sodium hydroxide (≥97.0%) obtained from Carlo erba.

### 3.3. Deep Eutectic Solvent Preparation

The Deep Eutectic Solvents (DESs) were prepared based on previously reported methodologies with some changes [55,79]. The DESs made in a molar ratio of two different components consisting of hydrogen bond donor (HBD) and hydrogen bond acceptor (HBA), followed by the addition of 20, 40, 60, and 80% of distilled water (Table 1). Sodium acetate used as HBA [79] and formic acid as HBD. Another DES prepared in the molarity ratio of sodium acetate and formic acid (1:2). The molarity ratio-based DES formulated due to the fact that the anhydrous mixtures are very viscous, difficult to manipulate [55], and unsuitable for polyphenolics extraction. The molar ratio mixture prepared by mixing the molar mass of components as gram in appropriate ratios. The molarity ratio mixture prepared by mixing ingredients as molarity in the proper ratios. In this work, DES components were placed in reaction flask at 75 °C with constant stirring for 2 h 30 min to obtain a homogeneous liquid. The DESs prepared in molar and molarity ratios were encoded as SAFA_0_, and SAFA_m_, respectively.

### 3.4. Characterization of DESs

#### 3.4.1. Rheology

The viscosity of DESs was measured using a Rheometer (Buchi, CH-9230 Flawil 1, Switzerland) fitted with a parallel geometry with 20 mm of diameter and gap 1 mm. The measurements carried out as described in [55].

#### 3.4.2. Fourier Transformed Infrared (FTIR)

FTIR analysis of DESs and extracts was carried out using a FTIR Spectrometer (Perkin Elmer, Spectrum-Two, USA, PEService 35). Diamond lens attenuated resistance was used. The spectrometer was adjusted in resolution 4 and by selecting the Norton-Beer (*n*-B) strong apodization function. The range of all spectra was between the wavenumbers of 4000 and 400 cm^−1^. Prior to every spectrum, a background reference was taken using an empty cell to ensure no interferences. Then, spectrum intensity was transformed into relative transmittance, %T.

### 3.5. Extraction with DES and Conventional Solvents

Ultrasound-assisted extractions (UAE) performed in a sonication water bath (WUC-A03H, daihan scientific Co., Ltd. Seoul, Korea). Distilled water, 70% ethanol, and methanol were used as conventional solvents as they have exhibited high extraction performance of anthocyanins from Roselle [6,14,16,17]. 0.5 g of comminuted Roselle calyces added with 20 mL of conventional solvents and DESs. The mixture was ultrasonicated in an ultrasonic bath at room temperature (25 °C) for 20 min. The samples were left to cool and then filtered.

### 3.6. Optimization Parameters of DES and Extraction with Response Surface Methodology

The polyphenolic yield is affected by different operational factors such as temperature, time, liquid-solid ratio, the molar ratio of DES, speed of agitator and particle size. Herein, only three of them examined: Liquid-to-solid ratio, molarity ratio, and additional water content. The DES that provides the highest yield of anthocyanin was selected for the optimization process.

The optimization parameters of the DES examined systematically using response surface methodology based on the three-level Box-Behnken design (Design expert software 9.0). The experimental design carried out with three independent variables of X_1_ (molarity ratio), X_2_ (additional water content), and X_3_ (solvent to solid ratio). The actual and coded values of the independent variables presented in Table 7. The combinations of the molarity ratio of sodium acetate (1) to formic acid (1, 2.5 and 4), additional water (0%, 30% and 60%) and solvent to solid ratio (10:0.5, 25:0.5 and 40:0.5 mL/g) were independent variables chosen for UAE. These variables regrouped in 17 experimental points, including five replicates at the central point. Total anthocyanin, total phenolic content, FRAP, DPPH radical scavenging activity, and total flavonoid values investigated as the responses (Y) for the design experiment. The experimental points, together with responses, were displayed in Table 3. The experimental data fitted to the following quadratic polynomial model:

(6) $$Y=\;\beta 0+\;\sum_{i=1}^{3}\beta iXi+\sum_{i=1}^{3}\beta iiXii\;+\sum_{i=1}^{2}\sum_{j=i+1}^{3}\beta ijXiXj$$

### 3.7. Determination of Total Phenolic Content (TPC)

The TPC of Roselle extracts was determined using the Folin-Ciocalteu method [51,60]. A UV-spectrophotometer (Thermo Spectronic) used to read absorbance at 760 nm, and the TPC in each extract was calculated from a calibration curve (Y = 0.0009x − 0.0125; R^2^ = 0.9977), using gallic acid as a standard. The results were given as mg gallic acid equivalent (GAE) g^−1^ dw.

### 3.8. Determination of Total Flavonoid (TFC)

The total flavonoid was determined using a modified protocol [51,57,80]. The absorbance was measured at 510 nm after 10 min in the dark at room temperature. The TFC was calculated from a calibration curve using epicatechin as standard (Y = 17.062x + 0.0152; R^2^ = 0.9994). The results estimated as mg epicatechin equivalents (ECE) g^−1^ dw.

### 3.9. Determination of Total Anthocyanin (TACN)

A modified pH differential method [48] was employed to quantify the total anthocyanin of Roselle calyces. Briefly, 0.05 mL aliquot of the extract diluted with 1.95 mL of buffer (consisting of 1.86 g of KCl, 980 mL of distilled water and 6.3 mL HCl), pH 1.0 and another 0.05 mL aliquot of the extract diluted with 1.95 mL of buffer, (consisting of 54.43 g of sodium acetate, 960 mL distilled water and 20 mL HCl) solution pH 4.5. The buffer solutions completed up to 1 L with distilled water. Afterward, the absorbance measured at 518 nm, and the total anthocyanin content was calculated in function of mg delphinidin-3-sambubioside equivalent (D3S E) g^−1^ with the following equation,

(7) $$TACN\;\left(mg/kg\right)=\frac{A_{t}\times M_{w}\times D_{f}\times 1000}{\varepsilon \times l}$$

where A = (Absorbance_518 nm_ − Absorbance_700 nm_) pH 1.0 − (Absorbance_518 nm_ − Absorbance_700 nm_) pH 4.5; M_W_ (molecular weight) = 571 g·mol^−1^ for D3S, DF = dilution factor established in D, l = pathlength in cm and *Ԑ* = 26,900 molar extinction coefficient, in L × mol^−1^ × cm^−1^, for delphinidin-3-sambubioside.

### 3.10. Determination of Ferric Reducing Antioxidant Power (FRAP)

FRAP assay performed according to the procedure of [62,80]. The FRAP values of the extracts were calculated from the calibration curve (Y = 0.8325 − 0.0936; R^2^ = 0.9966) using FeSO_4_ as a standard. The results were given as mmol FeSO_4_ equivalents (mmol ISE g^−1^dw) [81].

### 3.11. Determination of the DPPH Radical Scavenging Activity

DPPH radical scavenging activity determined according to previous studies [51,82,83]. An aliquot of 50 μL of the sample added with 1 mL DPPH solution (0.06 mM in 80% methanol). The absorbance at 517 nm was recorded. The DPPH solution used as control. The reduction ratio of DPPH determined with the following equation,

(8) $$\mathrm{Reduction}\;\left(\%\right)=\left(\frac{A_{c}-A_{s}}{A_{c}}\right)\times 100$$

where Ac = Absorbance of control and As = Absorbance of extract. The DPPH radical scavenging activity in each extract was calculated from a calibration curve (Y = 149.11x − 0.7773; R^2^ = 0.9977) considering the reduction ratio as Y and using Trolox as a standard. The results were given as mmol Trolox equivalent (TE) g^−1^ dw [84].

### 3.12. Stability Tests

#### 3.12.1. Thermal Stability

For the thermal stability, 10 mL of Roselle extracts were put in the brown bottles with screw caps and placed in a preheated water bath at 100, 80, 60 and 40 °C. Three bottles of each group were removed from the water bath after 20, 40, 60, 80 and 100 min and cooled to room temperature. To evaluate the kinetic modeling of TPC, TFC, TACN, DPPH radical scavenging and FRAP values of DES extracts of Roselle, the first-order reaction rate constant (k) calculated [58,85],

(9) $$\ln(\frac{C_{t}}{C_{0}})=-\mathrm{kt}$$

where k is the constant rate (s^−1^), C_0_ is the initial concentration and C_t_ is the concentration after the heating time (t) at a given temperature.

The kinetics of thermal degradation of anthocyanin of Roselle was evaluated with parameter activation energy (Ea) which was determined as described in [74],

(10) $$\frac{k}{k_{ref}}=exp^{\left(\frac{-Ea}{R}\left(\frac{1}{T}\;-\;\frac{1}{T_{ref}}\right)\right)}\;$$

where k is the constant rate (s^−1^), k_ref_ is the constant rate (s^−1^) of a reference temperature T_ref_ (K). 40 °C considered as reference temperature in the present study. Ea is the activation energy (J mol^−1^) and R is the universal gas constant (8.32 J mol^−1^ K^−1^).

#### 3.12.2. Storage Stability

The effect of storage time was investigated at −20 °C, 4 °C, and ambient conditions in the dark. 10 mL of Roselle extracts were put in brown bottles and placed in the dark at −20 °C, 4 °C and ambient temperature (20 °C). Three bottles of each group were removed and analyzed at days 0, 3, 7, 15 and 18. The first-order rate constant (k) and half-life time (t_1/2_) used to determine the kinetic modeling of anthocyanin degradation during the storage [14,58]:

(11) $$t_{1/2}=\frac{-\ln\left(0,5\right)}{k}$$

### 3.13. Data and Statistical Analyses

All studies were performed in triplicates and the mean values were determined. The software Design Expert 9.0 (Trial version, Stat-Ease Inc., Mineapolis, MN USA) was used to design the experimentation along with data analysis. ANOVA was used to determine the statistical relationship between factors. The adequacy of the models obtained was ascertained by screening the R^2^, adjusted R^2^, coefficient of variation (CV) and the value of Fisher’s test (F-value). The significance of the models and regression coefficients were measured at *p* < 0.05. The relationship between independent variables and responses was checked by 3D graphics. The optimum conditions were determined according to the desirability function. One-way statistical analyses were carried out by ANOVA with post-hoc Duncan’s test using SPSS (version 21). The significance of the results was assessed at *p* ≤ 0.05.

## 4. Conclusions

The analysis of the effect of water addition revealed that the viscosity and DES nanostructure decreased upon water addition. However, the extraction of phytochemical compounds and the antioxidant properties of Roselle increased considerably after the water introduction. SAFA_m_ with 1:2 molarity ratio was revealed to be the most efficient for the extraction of anthocyanins and polyphenol antioxidants from Roselle calyces when compared to SAFA_0_, SAFA_20_, SAFA_40_, SAFA_60_, SAFA_80_ and conventional solvents. Subsequently, this DES was selected for the optimization using a Box-Behnken design paired with response surface methodology to determine the optimum point for the extraction of maximum anthocyanins from Roselle. The optimum point was determined as 1:3.6 molarity ratio, 0% additional water and 10 mL solvent ratio. Under these optimum conditions, TPC, TFC, TACN, FRAP and DPPH radical scavenging were found to be 233.26 mg GAE/g, 10.14 mg ECE/g, 10.62 mg D3S/g, 493.45 mmol ISE/g, and 343.41 mmol TE/g, respectively. The thermal and storage tests performed on Roselle extracts showed that the phytochemical compounds mainly anthocyanins were more stable in SAFA_m_. This novel DES represented a green and sustainable solvent for the extraction of bioactive compounds.

## Figures and Tables

**Table 1:** DESs preparation and corresponding viscosities.

DES.	Water Content %	Molar Ratio	Molarity Ratio	Viscosity (mPa.s)
SAFA_0_	0	1:2	-	3651.60 ± 14.60a
SAFA_20_	20	1:2	-	61.60 ± 5.98b
SAFA_40_	40	1:2	-	17.36 ± 0.79c
SAFA_60_	60	1:2	-	10.81 ± 0.15c
SAFA_80_	80	1:2	-	7.66 ± 0.46c
SAFA_m_	-	-	1:2	6.15 ± 1.27c

Different letters (a, b, c) in the same column indicate statistical differences (*p* < 0.05).

**Table 2:** TPC, TFC, TACN and antioxidant activity (DPPH radical scavenging and FRAP) of Roselle calyces using DES and conventional solvents.

Solvents.	TPC, mg GAE/g	TFC, mg ECE/g	TACN, mg D3S/g	DPPH Radical Scavenging, mmol TE/g	FRAP, mmol ISE/g
SAFA_0_	6.95 ± 0.41d	0.44 ± 0.07e	0.13 ± 0.03e	5.84 ± 0.93g	58.99 ± 3.42c
SAFA_20_	141.30 ± 8.53c	2.28 ± 0.13d	3.93 ± 0.23d	52.55 ± 5.78f	435.61 ± 31.04a
SAFA_40_	198.49 ± 3.22b	3.57 ± 0.38c	5.27 ± 0.56c	60.62 ± 20.11ef	448.99 ± 57.01a
SAFA_60_	199.83 ± 10.54b	3.71 ± 0.29c	5.42 ± 0.91c	82.22 ± 5.55de	465.98 ± 29.16a
SAFA_80_	202.17 ± 4.37b	3.75 ± 0.28c	5.85 ± 0.29bc	87.27 ± 6.80d	484.06 ± 2.73a
SAFA_m_	248.26 ± 26.99a	10.42 ± 0.15a	7.01 ± 0.04a	372.34 ± 9.56a	451.20 ± 1.58a
distilled water	141.23 ± 17.96c	2.09 ± 1.04d	6.44 ± 0.20ab	313.41 ± 13.91c	391.91 ± 9.33b
70% ethanol	141.11 ± 24.14c	8.55 ± 0.83b	6.80 ± 0.68a	354.97 ± 27.41ab	450.86 ± 7.76a
80% methanol	139.83 ± 32.38c	2.95 ± 0.19cd	5.68 ± 0.46bc	337.42 ± 15.40bc	467.75 ± 21.76a

Different letters (a, b, c, d, e, f, g) in the same column indicate statistical differences (*p* < 0.05).

**Table 3:** Coded Box-Behnken design with the analytical responses.

Run	Coded Values			Analytical Responses				
	X_1_	X_2_	X_3_	Y_1_	Y_2_	Y_3_	Y_4_	Y_5_
1	0	+1	−1	285.36	187.65	387.09	8.54	8.12
2	0	0	0	225.09	115.48	318.29	8.54	7.49
3	−1	0	−1	253.27	218.65	413.77	6.71	8.45
4	0	+1	+1	295.17	180.24	350.69	3.78	8.34
5	0	0	0	230.54	105.81	305.86	8.41	6.80
6	0	0	0	231.89	121.86	325.44	7.31	7.12
7	+1	−1	0	343.76	233.20	493.88	10.13	10.90
8	+1	+1	0	297.80	206.55	395.52	8.95	7.5
9	0	−1	+1	307.40	206.40	420.22	6.08	9.5
10	0	−1	−1	339.30	265.03	466.24	8.78	8.74
11	0	0	0	222.05	105.83	310.74	8.12	7.03
12	+1	0	+1	255.15	213.36	371.96	4.41	8.46
13	−1	−1	0	276.96	211.34	396.57	9.01	8.49
14	0	0	0	230.54	205.30	315.35	7.56	6.50
15	+1	0	−1	327.05	127.11	423.06	9.02	8.5
16	−1	0	+1	279.46	177.04	339.79	4.37	8.30
17	−1	+1	0	280.89	180.32	398.58	7.34	8.50

X_1_ (Molarity ratio); X_2_ (Additional water, %) and X_3_ (Solvent ratio, mL). Y_1_ (DPPH radical scavenging, mmol TE/g); Y_2_ (TPC, mg GAE/g); Y_3_ (FRAP, mmol ISE/g); Y_4_ (TFC, mg ECE/g) and Y_5_ (TACN, mg D3S/g).

**Table 4:** ANOVA results for the reduced quadratic models.

	TPC			TFC			TACN			DPPH Radical Scavenging			FRAP		
	SS	F-Value	*p*-Value	SS	F-Value	*p*-Value	SS	F-Value	*p*-Value	SS	F-Value	*p*-Value	SS	F-value	*p*-Value
model	41,224.33	63.93	<0.0001	57.49	30.84	<0.0001	16.54	21.77	0.0003	25,303.24	56.28	<0.0001	48,608,31	32.78	<0.0001
X_1_	526.91	7.35	0.0301	-	-	-	2.07	24.51	0.0017	1919.57	38.42	0.0004	-	-	-
X_2_	10,419.57	145.42	<0.0001	-	-	-	3.15	37.30	0.0005	10,107.63	202.33	<0.0001	13,644.21	82.80	<0.0001
X_3_	10,289.55	143.61	<0.0001	20.17	97.35	<0.0001	0.7286	8.63	0.0218	3544.30	70.95	<0.0001	2683.79	16.29	0.0050
X_12_	-	-	-	-	-	-	3.28	38.80	0.0004	625.00	12.51	0.0095	2500.00	15.17	0.0059
X_13_	-	-	-	2.25	10.86	0.0132	-	-	-	2401.00	48.06	0.0002	-	-	-
X_23_	676.00	9.43	0.0180	2.25	10.86	0.0132	-	-	-	441.00	8.83	0.0208	-	-	-
X_11_	9812.53	136.95	<0.0001	-	-	-	2.20	26.03	0.0014	1982.69	39.69	0.0004	7903.39	47.96	0.0002
X_22_	10,119.79	141.24	<0.0001	2.06	9.96	0.0160	4.84	57.35	0.0001	10,400.38	208.19	<0.0001	16,486.87	100.05	<0.0001
X_33_	10,642.42	148.53	<0.0001	17.69	85.42	<0.0001	1.61	19.01	0.0033	3590.06	71.86	<0.0001	3438.02	20.86	0.0026
residual	501.55			1.45			0.59			349.70			1153.45		
lack of fit	280.75	1.70	0.3046	0.25	0.28	0.84	0.40	2.77	0.17	288.50	6.29	0.0540	920.25	5.26	0.0713
total	41,725.88			58.94			17.13			25,652.94			49,761.76		
R^2^	0.9880			0.9754			0.9655			0.9864			0.9768		
adjusted R^2^	0.9725			0.9438			0.9211			0.9688			0.9470		
predicted R^2^	0.8841			0.9003			0.6098			0.8163			0.6968		
adequate precision	22.75			20.41			15.84			21.06			18.13		
C.V. %	4.71			6.45			3.63			2.57			3.40		

SS: Sum of squares.

**Table 5:** Effect of temperature and time on the k and Ea values of anthocyanin degradation kinetics of Roselle extracted with DES.

Time (min)	K				Ea (kJ/mol)
	40 °C	60 °C	80 °C	100 °C	
20	2.39 × 10^−5^ ± 3.73 × 10^−5^f	11.92 × 10^−5^ ± 6.32 × 10^−5^d	9.00 × 10^−5^ ± 0.94 × 10^−5^de	49.96 × 10^−5^ ± 5.52 × 10^−5^a	49.23 ± 16.46a
40	2.58 × 10^−5^ ± 1.20 × 10^−5^f	6.48 × 10^−5^ ± 0.39 × 10^−5^ef	5.05 × 10^−5^ ± 4.63 × 10^−5^ef	33.82 × 10^−5^ ± 0.65 × 10^−5^c	41.72 ± 7.16a
60	3.42 × 10^−5^ ± 0.78 × 10^−5^f	5.05 × 10^−5^ ± 0.70 × 10^−5^ef	6.20 × 10^−5^ ± 1.48 × 10^−5^ef	33.68 × 10^−5^ ± 4.40 × 10^−5^c	37.07 ± 2.11a
80	3.39 × 10^−5^ ± 0.72 × 10^−5^f	4.63 × 10^−5^ ± 2.21 × 10^−5^ef	7.14 × 10^−5^ ± 1.01 × 10^−5^def	32.79 × 10^−5^ ± 2.31 × 10^−5^c	36.78 ± 2.35a
100	3.12 × 10^−5^ ± 0.78 × 10^−5^f	6.67 × 10^−5^ ± 1.27 × 10^−5^ef	7.56 × 10^−5^ ± 1.45 × 10^−5^def	41.66 × 10^−5^ ± 2.88 × 10^−5^b	41.97 ± 4.60a

Different letters (a, b, c, d, e, f) indicate statistical differences (*p* < 0.05).

**Table 6:** Effect of storage on the k and t_1/2_ values of anthocyanin degradation kinetics of Roselle extracted with DES.

Temperature (°C)	3 Days		6 Days		9 Days		12 Days		15 Days		18 Days	
	k	t_1/2_	k	t_1/2_	k	t_1/2_	k	t_1/2_	K	t_1/2_	k	t_1/2_
20	4.34 × 10^−7^ ± 5.34 × 10^−8^a	1.61 × 10^6^ ± 1.92 × 10^5^b	7.41 × 10^−7^ ± 3.29 × 10^−8^a	0.94 × 10^6^ ± 0.43 × 10^5^c	4.59 × 10^−7^ ± 1.55 × 10^−8^a	1.51 × 10^6^ ± 0.50 × 10^5^b	4.34 × 10^−7^ ± 1.22 × 10^−8^a	1.60 × 10^6^ ± 0.45 × 10^5^c	3.95 × 10^−7^ ± 3.01 × 10^−9^a	1.87 × 10^6^ ± 1.75 × 10^5^b	2.73 × 10^−7^ ± 1.72 × 10^−8^a	2.31 × 10^6^ ± 2.54 × 10^5^b
4	2.63 × 10^−7^ ± 0.96 × 10^−8^b	2.63 × 10^6^ ± 0.98 × 10^5^a	3.31 × 10^−7^ ± 2.82 × 10^−8^b	1.11 × 10^6^ ± 1.85 × 10^5^b	1.51 × 10^−7^ ± 2.82 × 10^−8^b	4.65 × 10^6^ ± 5.34 × 10^5^a	2.00 × 10^−7^ ± 7.31 × 10^−9^b	3.47 × 10^6^ ± 1.28 × 10^5^b	1.65 × 10^−7^ ± 2.14 × 10^−8^b	4.25 × 10^6^ ± 5.72 × 10^5^a	1.47 × 10^−7^ ± 2.34 × 10^−8^b	4.49 × 10^6^ ± 8.12 × 10^5^a
−20	2.52 × 10^−7^ ± 3.33 × 10^−8^b	2.78 × 10^6^ ± 3.69 × 10^5^a	2.38 × 10^−7^ ± 3.52 × 10^−8^c	2.96 × 10^6^ ± 4.43 × 10^5^a	1.29 × 10^−7^ ± 1.15 × 10^−8^b	5.39 × 10^6^ ± 0.81 × 10^5^a	1.41 × 10^−7^ ± 2.72 × 10^−8^c	5.05 × 10^6^ ± 10.91 × 10^5^a	1.57 × 10^−7^ ± 1.01 × 10^−8^b	4.42 × 10^6^ ± 2.92 × 10^5^a	1.38 × 10^−7^ ± 1.01 × 10^−8^b	5.06 × 10^6^ ± 3.55 × 10^5^a

Different letters (a, b, c) in the same column indicate statistical differences (*p* < 0.05). k (s^−1^) and t_1/2_ (s).

**Table 7:** Actual and coded values of independent variables.

Coded Values	Actual Values		
	X_1_	X_2_	X_3_
−1	1:1	0	10
0	1:2.5	30	25
+1	1:4	60	40

X_1_ (Molarity ratio); X_2_ (Additional water, %) and X_3_ (Solvent ratio, mL).
